# Bending‐Induced Vibrational Landscape Reorganization Governs Energy Dissipation in Perylene Bisimides

**DOI:** 10.1002/anie.1926167

**Published:** 2026-06-26

**Authors:** Wei Zhang, Di Zhao, Byeongjoo Kang, Hui‐Jun Zhang, Yeonju Park, Young Mee Jung, Woojae Kim, Jianbin Lin, Dongho Kim

**Affiliations:** ^1^ Spectroscopy Laboratory for Functional π‐Electronic Systems and Department of Chemistry Yonsei University Seoul Republic of Korea; ^2^ Department of Chemistry College of Chemistry and Chemical Engineering Xiamen University Xiamen P. R. China; ^3^ Department of Chemistry Yonsei University Seoul Republic of Korea; ^4^ Department of Chemistry Institute For Molecular Science and Fusion Technology and Kangwon Radiation Convergence Research Support Center Kangwon National University Chuncheon Republic of Korea

**Keywords:** energy dissipation, internal conversion, time‐resolved impulsive stimulated Raman spectroscopy, vibrational coherence

## Abstract

Structural distortion is widely recognized to suppress emissions in organic chromophores; however, the mechanistic origin of the associated enhancement in nonradiative decay remains unresolved. Here, we employ a series of perylene bisimide derivatives with systematically controlled bending to directly elucidate the structural origin of distortion‐enhanced nonradiative relaxation. A pronounced transition is observed from near‐unity emission to strongly enhanced nonradiative decay within the singlet manifold, while intersystem crossing remains negligible. Time‐resolved electronic spectroscopy excludes triplet‐mediated pathways and establishes internal conversion as the dominant decay channel. Crucially, time‐resolved Raman measurements provide direct insight into the underlying structural dynamics, revealing that bending suppresses the electronically coupled aromatic skeletal mode, reorganizes the low‐frequency vibrational manifold, and accelerates vibrational dephasing. The results herein demonstrate that bending enhances internal conversion not primarily through energy‐gap reduction, but by transforming the excited‐state vibrational landscape into a more dissipative environment that facilitates efficient energy relaxation. More broadly, this work identifies vibrational dissipation and coherence loss as key determinants of nonradiative decay and establishes a general structure–vibrational dynamics framework for controlling excited‐state energy flow in π‐conjugated systems.

## Graphical abstract

Gradually bent perylene bisimides (PBIs) isolate structural distortion as the sole variable and exhibit negligible intersystem crossing, uncovering rapid vibrational dephasing as the dominant driver of internal conversion through excited‐state vibrational landscape reorganization.

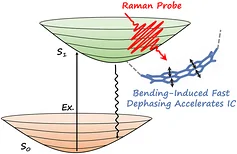

## 1 Introduction

Excited‐state relaxation dictates the fate of absorbed energy in molecular systems, determining whether excitation is emitted or dissipated [1]. The outcome arises from competition between radiative and nonradiative pathways and is acutely sensitive to molecular geometry [2]. Even subtle structural distortions can reshape excited‐state potential energy surfaces, perturb electronic transition properties, and reconfigure vibronic coupling [3, 4]. Among nonradiative channels, internal conversion (IC) provides a dominant pathway for funneling electronic excitation into vibrational degrees of freedom [5, 6]. A mechanistic description of IC therefore requires an explicit account of nuclear motion. Although structural distortions—such as bending, torsion, and out‐of‐plane deformation—are widely recognized to enhance nonradiative decay [7], the physical origin of this effect remains unresolved. In particular, it is unclear whether distortion promotes IC primarily through electronic effects, including energy‐gap modulation and changes in electronic transition properties, or through reorganization of the vibrational manifold that governs energy dissipation. Disentangling these contributions is essential for establishing a predictive framework linking molecular structure to excited‐state energy flow.

Perylene bisimide (PBI) derivatives provide a uniquely well‐controlled platform to address this question. The rigid π‐conjugated scaffold, intense π–π* transitions, and synthetically tunable substitution patterns enable systematic structural perturbation while largely preserving the underlying electronic framework [8, 9, 10, 11, 12, 13, 14]. Bending of the PBI core induces pronounced bathochromic shifts, spectral broadening, and substantial modulation of emission properties [15, 16, 17, 18, 19, 20]. The changes are typically attributed to reduced planarity and perturbed conjugation; however, such interpretations remain largely phenomenological and do not resolve the underlying relaxation mechanism. Despite extensive studies of distorted PBI systems [19, 20, 21], a molecular‐level understanding of how structural distortion governs excited‐state relaxation remains lacking. In particular, direct identification of the vibrational coordinates that mediate energy dissipation has remained elusive, limiting the development of general structure–dynamics relationships in π‐conjugated chromophores.

A central challenge is that conventional spectroscopies primarily track electronic populations and relaxation kinetics, whereas the nuclear motions underlying IC remain largely inaccessible. While transient absorption and time‐resolved fluorescence measurements define relaxation pathways and timescales, they do not directly resolve the vibrational coordinates responsible for nonradiative decay. This limitation is especially acute in structurally distorted systems, where nuclear motion is expected to dominate energy dissipation [6, 22]. Time‐resolved impulsive stimulated Raman spectroscopy (TR‐ISRS) provides direct access to coherent vibrational motion on excited‐state potential energy surfaces and is therefore uniquely suited to interrogate vibrational contributions to IC [23, 24, 25]. A systematically bent PBI series is particularly advantageous in this context, as it enables controlled variation of molecular geometry without fundamentally altering the electronic framework, thereby isolating structure‐induced changes in vibrational dynamics.

Here, we employ a series of PBI derivatives with systematically varied bending to resolve the origin of distortion‐enhanced nonradiative relaxation. We uncover a counterintuitive yet general trend in which increasing structural distortion suppresses radiative emission while markedly accelerating singlet‐state nonradiative decay, with negligible intersystem crossing (ISC). Crucially, this behavior cannot be rationalized by conventional energy‐gap arguments. Instead, bending induces a pronounced reorganization of the excited‐state vibrational landscape, characterized by suppression of electronically coupled aromatic skeletal modes, redistribution of low‐frequency structural modes, and accelerated vibrational dephasing. The changes collectively create a highly dissipative vibrational environment that promotes rapid loss of vibrational coherence and efficient energy redistribution. Our results establish vibrational landscape reorganization—rather than energy‐gap modulation—as the dominant mechanism governing distortion‐enhanced IC. More broadly, this work defines a structure–vibrational dynamics paradigm for excited‐state relaxation and identifies geometric distortion as a powerful and general strategy for controlling energy dissipation in π‐conjugated systems.

## 2 Results and Discussion

### 2.1 Steady‐State Optical Properties

**FIGURE 1:**
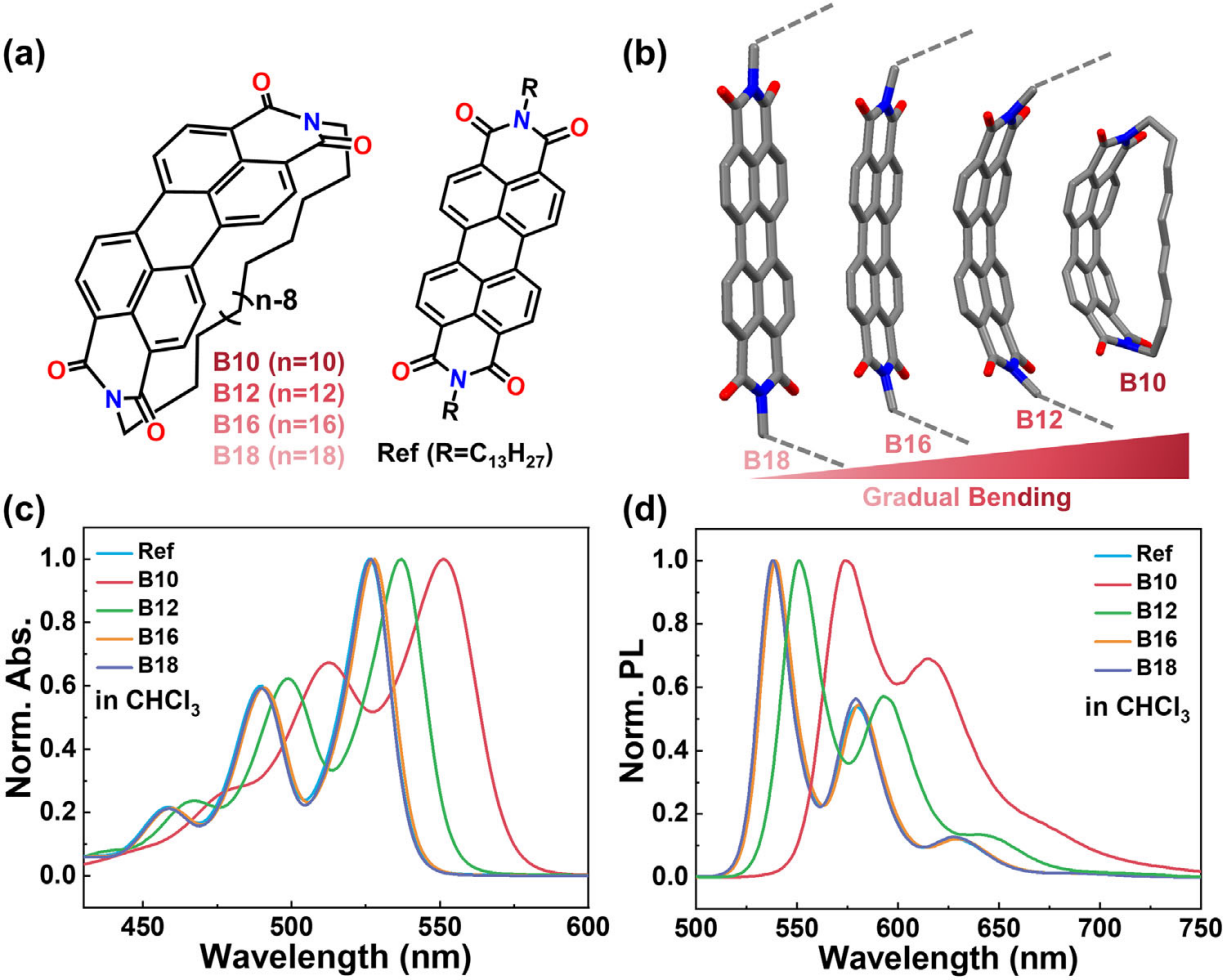
(a) Molecular structures of the end‐to‐end bent PBI cycloalkanes **B10**, **B12**, **B16**, and **B18**, together with the planar PBI monomer (**Ref**). (b) The optimized ground‐state geometries of **B10**‐**B18**. (c) Steady‐state absorption spectra and (d) PL spectra of the PBI derivatives. The Norm. Abs. means normalized absorption, Norm. PL stands for normalized photoluminescence.

The PBI derivatives were synthesized via a modular sequence comprising imide condensation, Ni(0)‐mediated intramolecular Yamamoto coupling, and subsequent cyclodehydrogenation, following established procedures [21]. Systematic variation of the alkyl tether linking the two imide nitrogen atoms yields a series of PBI derivatives with progressively increasing structural bending, denoted as **B10**, **B12**, **B16**, and **B18** (Figure 1a). This design enables controlled modulation of geometric constraint while largely preserving the intrinsic electronic framework of the PBI core. Density functional theory (DFT) calculations were performed to optimize ground‐state geometries (Figure S1). As shown in Figure 1b, **B18** adopts an essential planar conformation comparable to the reference PBI monomer (denoted as **Ref**, see Figure 1a), whereas progressive shortening of the linker induces increasing deviation from coplanarity, with **B10** representing the most strongly bent structure in the series. To quantitatively evaluate the degree of structural bending, the curvature radius of the PBI core (Figure S2) was estimated from the optimized ground‐state geometries (as detailed in Supporting Information). The curvature radius (Table S1) increases from 6.98 Å for **B10** to 10.85 Å for **B12** and 65.74 Å for **B16**, whereas **B18** and **Ref** are nearly planar with an essentially infinite radius of curvature. This quantitative metric confirms the gradual reduction of structural bending from **B10** to **B18**. The combination of a conserved electronic backbone and systematically tunable geometry thus establishes a well‐defined platform for isolating the effect of structural bending on optical properties and excited‐state relaxation, ultimately enabling direct interrogation of structure‐controlled energy dissipation.

Steady‐state optical spectra reveal a pronounced and systematic evolution of the electronic transition with increasing bending. In chloroform (CHCl_3_), **Ref** and the least distorted derivative **B18** exhibit intense and well‐resolved absorption bands characteristic of a rigid π‐conjugated framework (Figure 1c). Increasing structural distortion from **B18** to **B10** leads to progressive bathochromic shifts and spectral broadening, accompanied by a substantial decrease in molar absorption coefficient (ε, see Table 1). Specifically, the ε (Figure S3) decreases monotonically from 7.94 × 10^4^ L mol^−1^ cm^−1^ for **Ref** to 3.32 × 10^4^ L mol^−1^ cm^−1^ for **B10**, indicating attenuation of transition strength with loss of planarity. Consistently, calculated oscillator strengths decrease from 0.7856 (**Ref**) to 0.5825 (**B10**) (Table 1), reflecting a reduced vertical transition probability upon bending. Frontier molecular orbital (FMO) analysis (Figure S4) shows that the lowest‐energy excitation remains dominated by the highest occupied molecular orbital (HOMO) to lowest unoccupied molecular orbital (LUMO) transition (H→L) across the series (Table 1), confirming that the electronic character of the first excited state (S_1_) is preserved despite substantial changes in transition intensity. In addition, the computed ground state (S_0_) to S_1_ transition dipole moment decreases from 3.35 to 2.95 Debye (D) from **Ref** to **B10** (Table 1), in agreement with the observed reduction in oscillator strength and radiative rate. This trend is consistent with the structural interpretation proposed for related bent PBI systems [19], in which bending‐induced contraction along the long molecular axis was associated with reduced electronic transition strength. The results collectively demonstrate that structural bending weakens transition intensity while maintaining the fundamental electronic identity of the lowest excited state. This is further evidenced by the electron‐hole analysis (Figure S5), in which the electronic transition (S_0_→S_1_) are mainly localized on PBI core.

**TABLE 1:** The photophysical data of PBI derivatives.

Samples	τ^a^/ns	ε^b^/10^4^ L mol^−1^ cm^−1^	Δν^c^/cm^−1^	Φ_PL_ ^d^/%	*k_r_* ^e^, ^k^/10^8^ s^−1^	*k_nr_* ^f^, ^k^/10^6^ s^−1^	S_1_ ^g^/eV	FMOs^h^	f^i^	µ_eg_ ^j^/D
**Ref**	3.7 ± 0.1	7.94	426	99 ± 1	2.68 ± 0.08	2.70 ± 2.70	2.3293	H→L 97.5%	0.7856	3.352
**B18**	3.8 ± 0.1	6.84	388	98 ± 1	2.58 ± 0.07	5.26 ± 2.63	2.3264	H→L 97.4%	0.7809	3.368
**B16**	3.9 ± 0.1	7.05	420	97 ± 0	2.49 ± 0.06	7.69 ± 0.20	2.3233	H→L 97.4%	0.7575	3.312
**B12**	4.3 ± 0	4.21	473	80 ± 4	1.86 ± 0.09	46.51 ± 9.30	2.2807	H→L 97.5%	0.6602	3.113
**B10**	5.4 ± 0.2	3.32	757	61 ± 5	1.13 ± 0.10	72.22 ± 9.64	2.2041	H→L 97.7%	0.5825	2.954

^a^ S_1_ lifetime.

^b^ Absorption coefficient at the 0–0 absorption band.

^c^ Stokes shift.

^d^ PL quantum yield.

^e^ Radiative decay rate.

^f^ Nonradiative decay rate.

^g^ The S_1_ energy levels were estimated from the intersections of the normalized absorption and emission spectra.

^h^ The FMOs that contribute to the S_0_ to S_1_ transition.

^i^ Oscillator strength.

^j^ Transition dipole moment.

^k^ The error bar calculation is detailed in Supporting Information. For [a–g], the data was collected in CHCl_3_; for [h–j], the data was collected in gas phase (wb97xd/def2SVP).

Photoluminescence (PL) measurements exhibit an even stronger sensitivity to structural bending (Figure 1d). Across the series, the emission progressively redshifts and broadens, evolving from relatively well‐resolved vibronic structure in **Ref** and **B18** to featureless profiles in the more strongly bent derivatives. Concomitantly, the Stokes shift increases substantially (Table 1), from 388–426 cm^−1^ (**Ref** and **B18**) to 757 cm^−1^ (**B10**), indicating enhanced geometrical reorganization between the absorbing and emitting states. The combined evolution of spectral shape and Stokes shift reflects increasing excited‐state structural relaxation with bending.

More strikingly, the bending dependence of PL quantum yield (PLQY, Φ_PL_) and decay kinetics (Figures S6 and S7) reveals a counterintuitive trend that motivates the central mechanistic question of this work. The Φ_PL_ (Table 1) decreases steadily with increasing bending, from near unity (**Ref** and **B18**) to 61% (**B10**), indicating the emergence of an additional nonradiative decay pathway that competes with photon emission. In contrast, the S_1_ lifetime increases from 3.7–3.8 ns (**Ref**, **B18**) to 5.4 ns (**B10**), implying a slower overall excited‐state decay despite reduced emission efficiency. Resolving this apparent inconsistency requires separation of radiative and nonradiative contributions. The radiative decay rate (*k_r_*, Table 1) decreases monotonically from 2.68 × 10^8^ s^−1^ (**Ref**) to 1.13 × 10^8^ s^−1^ (**B10**), consistent with reduced oscillator strength. In contrast, the nonradiative decay rate (*k_nr_*, Table 1) exhibits a much more pronounced and nonlinear increase, rising from 2.70 × 10^6^ s^−1^ (**Ref**) to 7.69 × 10^6^ s^−1^ (**B16**), followed by a sharp increase to 46.51 × 10^6^ (**B12**) and 72.22 × 10^6^ s^−1^ (**B10**). The results demonstrate that structural bending does not merely perturb spectral features but fundamentally redirects excited‐state energy flow toward nonradiative dissipation.

At first glance, the observed trend may appear consistent with the energy‐gap law [26, 27], which predicts enhanced nonradiative decay with decreasing S_1_/S_0_ energy separation due to increased density of accepting vibrational states. The progressive bathochromic shift and enlarged Stokes shift suggest a possible contribution from energy‐gap reduction. To evaluate this possibility more directly, the dependences of PLQY and *k_nr_* on S_1_ energy levels (Table 1) are further summarized in Figure S8. The results show that decreasing S_1_ energy levels are qualitatively accompanied by reduced PLQY and increased *k_nr_*, consistent with the general trend expected from the energy‐gap law. However, the sharp and nonlinear increase in *k_nr_* for the more strongly bent PBI derivatives indicates that energy‐gap reduction alone does not fully account for the pronounced enhancement of nonradiative decay. More importantly, the energy‐gap law assumes that the electronic character of the emitting state and the associated vibronic coupling framework remain essentially unchanged [27, 28, 29], such that variations in *k_nr_* can be primarily attributed to the changes in energy gap. This condition is not satisfied in the present system. Structural bending not only lowers the emission energy but also disrupts molecular planarity, modifies electronic transition properties, and amplifies excited‐state structural reorganization [6, 30, 31]. Consequently, the vibrational coordinates governing IC evolve systematically along the series and cannot be treated as invariant. The observed enhancement of nonradiative decay therefore reflects a more fundamental change in the excited‐state relaxation landscape beyond simple energy‐gap modulation alone.

A useful point of comparison is provided by recent studies of end‐to‐end bent PBI cyclophanes [19, 20]. In those systems, increased curvature similarly induced redshifted and broadened optical spectra but led to enhanced PLQYs and suppressed nonradiative decay. This contrasting behavior was attributed primarily to conformational restrictions imposed by the tethered architecture [19, 20], which limits structural relaxation of both the PBI core and substituents. In addition, interactions between the PBI core and heteroatom‐containing substituents may introduce additional electronic contributions, including possible charge‐transfer character, complicating the interpretation in terms of geometric effects alone. In contrast, the present series isolate the intrinsic consequence of structural bending, as the pronounced increase in *k_nr_* can be rationalized without invoking conformational restrictions or additional electronic interactions. This distinction highlights a key mechanistic question: how structural bending alone modulates excited‐state relaxation pathways in the absence of competing constraints or extrinsic electronic effects.

Taken together, the present photophysical results point to a bending‐activated mechanism that cannot be captured by conventional energy‐gap arguments. The pronounced increase in *k_nr_* indicates that structural distortion creates a progressively more dissipative excited‐state environment. This observation raises two critical questions. First, whether the enhanced nonradiative decay involves triplet formation via ISC. Second, if ISC is negligible which structural and vibrational factors promote increasingly efficient IC in the bent derivatives. To address these issues, time‐resolved electronic spectroscopic measurements were performed to identify the dominant excited‐state decay pathway.

### 2.2 Time‐Resolved Electronic Spectroscopy

Nonradiative decay in organic chromophores generally proceeds through multiple channels, including IC within the singlet manifold and ISC to triplet states followed by triplet relaxation [32]. To determine whether the bending‐induced increase in nonradiative decay arises from enhanced ISC, femtosecond and nanosecond transient absorption (fs‐ and ns‐TA) measurements were performed for representative PBI derivatives in degassed solutions.

The fs‐TA spectra of **B10**, **B12**, **B16**, and **B18** exhibit highly similar spectral features and temporal evolution (Figure S9), characterized by pronounced ground‐state bleach (GSB) and stimulated emission (SE) in the visible region, along with a broad excited‐state absorption (ESA) band extending into the red to near‐infrared (NIR) region. Notably, no additional photoinduced absorption bands unique to the more strongly bent derivatives are observed, and the early‐time dynamics remain comparable across the series. The observations indicate that structural bending does not introduce a new ultrafast branching pathway leading to a distinct intermediate on the femtosecond‐to‐nanosecond timescale.

**FIGURE 2:**
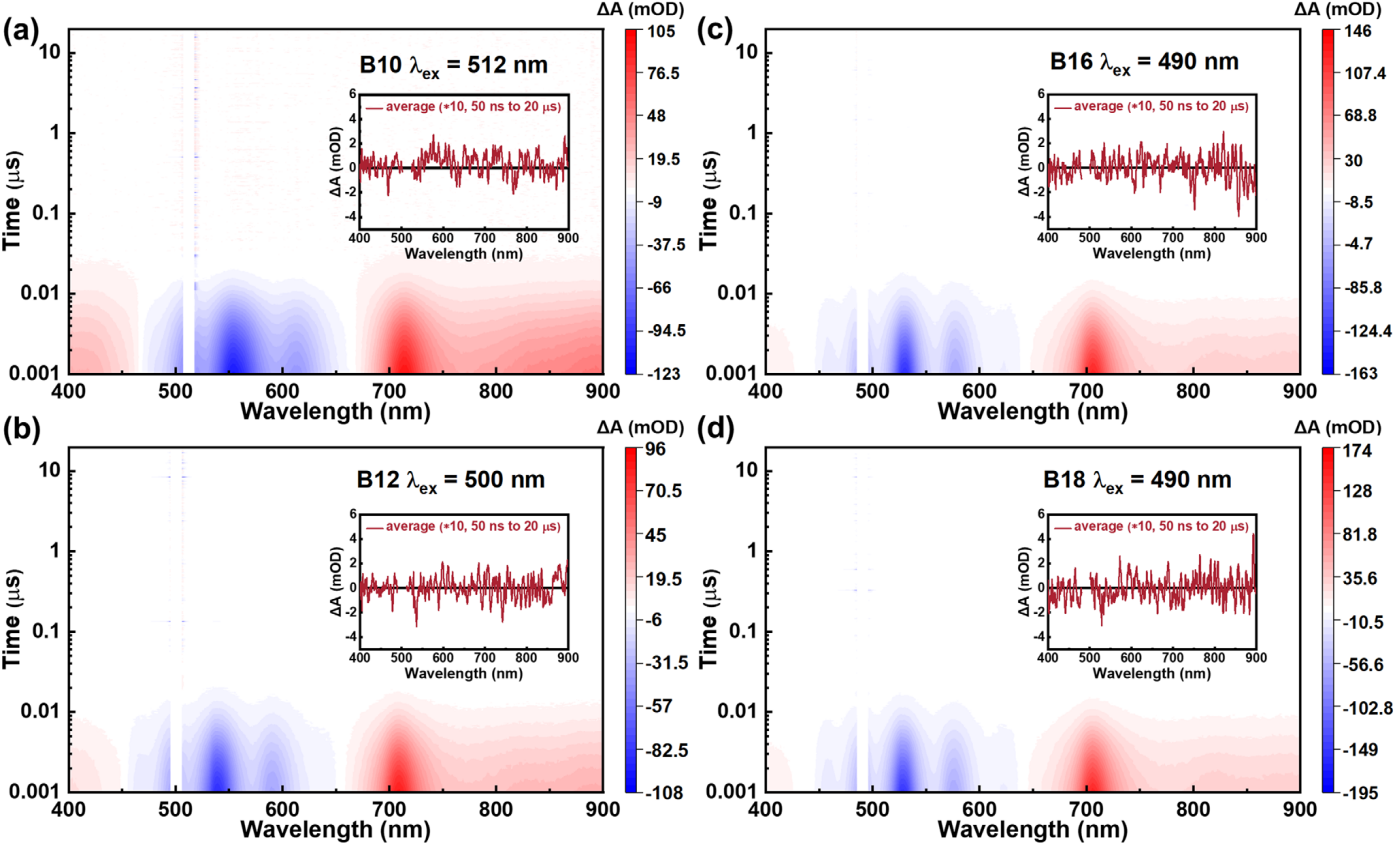
The ns‐TA spectra of (a) **B10**, (b) **B12**, (c) **B16**, and (d) **B18** in CHCl_3_. The inset in each figure is the enlarged averaged spectra of PBI derivatives. *λ*_ex_ stands for the excitation wavelength.

Ns‐TA measurements further constrain the contribution of triplet formation. The ns‐TA maps (Figure 2a–d) display only weak residual signals that decay toward baseline, without the emergence of a pronounced and persistent absorption feature characteristic of triplet excitons. Consistently, spectra averaged over long delay times (50 ns to 20 µs; insets in Figure 2a–d) remain near the noise level for all derivatives, indicating that the population of long‐lived species is negligible. Taken together, the fs‐ and ns‐TA results demonstrate that ISC contributes, at most, minimally to the bending‐enhanced nonradiative relaxation.

With ISC effectively excluded, the dominant origin of the bending‐amplified nonradiative decay is most plausibly attributed to IC from S_1_ to S_0_. The abrupt increase in nonradiative decay rate with bending, coupled with the concurrent growth of Stokes shift and spectral broadening, points to a distortion‐induced reconfiguration of the excited‐state relaxation landscape that facilitates more efficient energy dissipation within the singlet manifold. The absence of transient signatures associated with long‐lived intermediates further supports the conclusion that triplet‐mediated pathways are not operative. Collectively, the steady‐state and time‐resolved spectroscopic results identify IC as the primary nonradiative channel and implicate bending‐induced reorganization of the vibrational landscape as the key factor underlying its enhancement.

### 2.3 Steady‐ and Excited‐State Raman Spectroscopy

**FIGURE 3:**
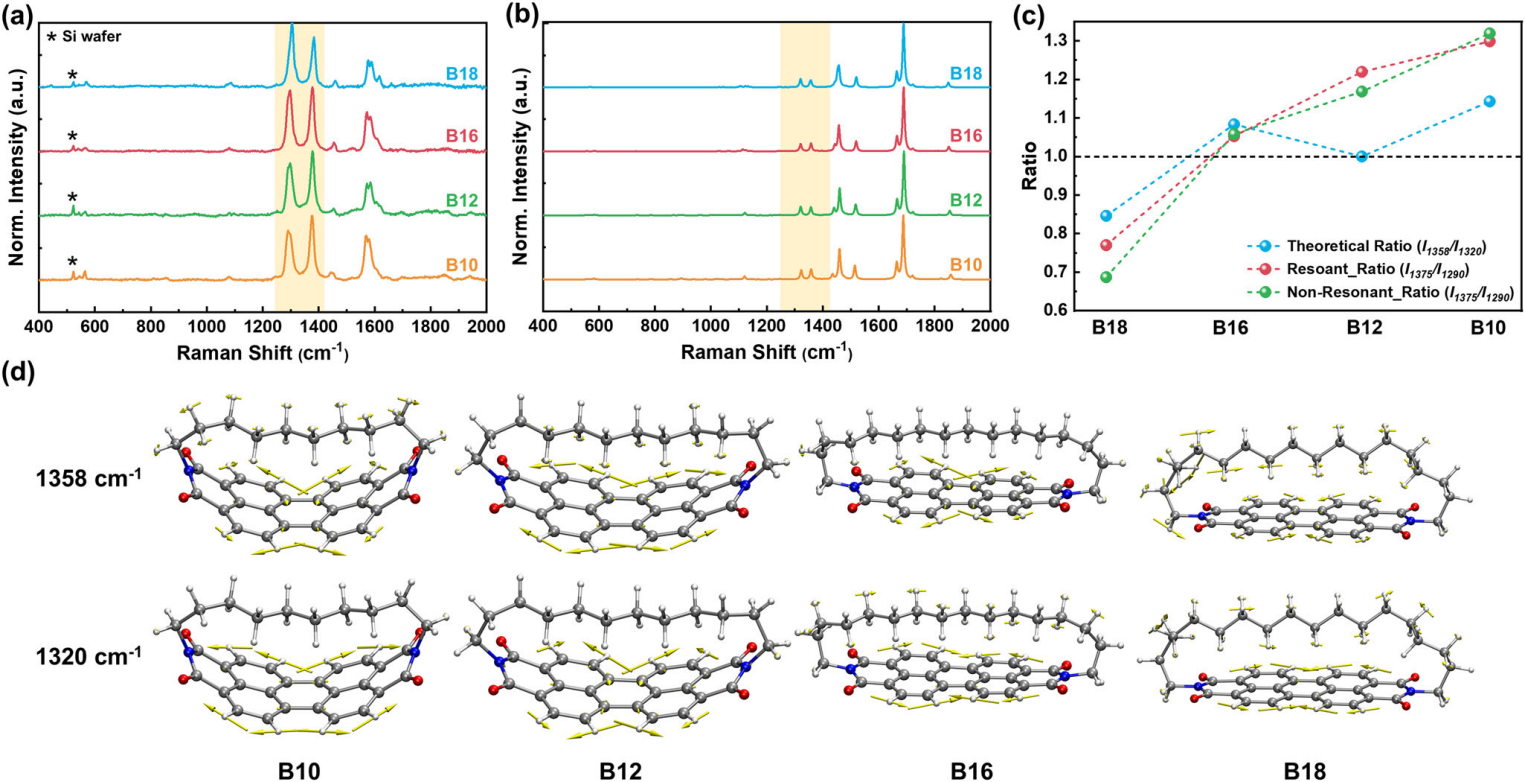
(a) The experimental ground‐state resonant Raman spectra of PBI derivatives with excitation at 473 nm. (b) The theoretical simulated ground‐state Raman spectra of PBI derivatives. (c) The Raman peak intensity ratios of the experimental and theoretical Raman data. (d) The vibrational modes of theoretically simulated ground‐state Raman of PBI derivatives.

To obtain direct structural insight into the origin of bending‐enhanced nonradiative decay, steady‐state Raman measurements were performed and compared with density functional theory (DFT)‐simulated spectra. The experimental resonant Raman spectra (Figure 3a) exhibit two prominent bands in the 1290 to 1375 cm^−1^ region whose relative intensities vary systematically across the series. For the more strongly bent derivatives (**B10**, **B12**, and **B16**), the higher‐frequency band near 1375 cm^−1^ dominates, whereas for the nearly planar **B18** derivative, the lower‐frequency band near 1290 cm^−1^ is more intense. Accordingly, the intensity ratio between the two modes (Figure 3c) reverses upon progressing from planar to bent structures, revealing a pronounced bending‐dependent redistribution of vibrational activity within the molecular framework.

A similar trend is observed in the non‐resonant Raman spectra (Figure S10), indicating that the intensity redistribution is intrinsic to structural distortion rather than arising from resonance enhancement effects. DFT‐calculated Raman spectra (Figure 3b) reproduce the experimental behavior well, with the corresponding modes appearing near 1320 cm^−1^ and 1358 cm^−1^ [33, 34]. In the calculations, the 1358 cm^−1^ mode is more intense than the 1320 cm^−1^ mode for **B10** and **B16**, whereas **B18** shows the opposite relation and **B12** lies intermediate between these limits. The close agreement between experiment and theory supports the reliability of the mode assignments and confirms that the observed intensity inversion originates from bending‐induced structural perturbation of the PBI framework.

Analysis of the calculated normal modes (Figure 3d) provides a structural basis for this behavior. In the planar **B18** structure, both modes involve coupled vibrational contributions from the aromatic PBI core and the alkyl tether, indicative of relatively delocalized vibrational character. With increasing bending, this coupling becomes progressively perturbed. In **B16**, the 1320 cm^−1^ mode retains mixed contributions, whereas the 1358 cm^−1^ mode becomes more strongly localized on the aromatic framework. In the more strongly bent derivatives (**B10** and **B12**), the vibrational character becomes increasingly differentiated, with the 1320 cm^−1^ mode primarily localized on the aromatic skeleton and the 1358 cm^−1^ mode involving mixed participation from both the aromatic core and the alkyl chain. Although the evolution is not strictly monotonic, the overall trend indicates that structural bending redistributes vibrational amplitude between the rigid π‐conjugated core and the flexible tether. To further clarify the bending dependence of the calculated Raman response, the Raman activities of representative high‐frequency aromatic C═C stretching modes are summarized in Table S2. The calculated activities increase from **B10** to **B18**, indicating that the intrinsic Raman response of the aromatic stretching modes is strengthened as the PBI framework becomes less distorted. This bending‐dependent reorganization of vibrational character is consistent with the optical trends described above, including progressive spectral broadening, increasing Stokes shifts and decreasing S_1_ energy levels, which collectively indicate enhanced structural relaxation and larger nuclear reorganization in the excited state.

**FIGURE 4:**
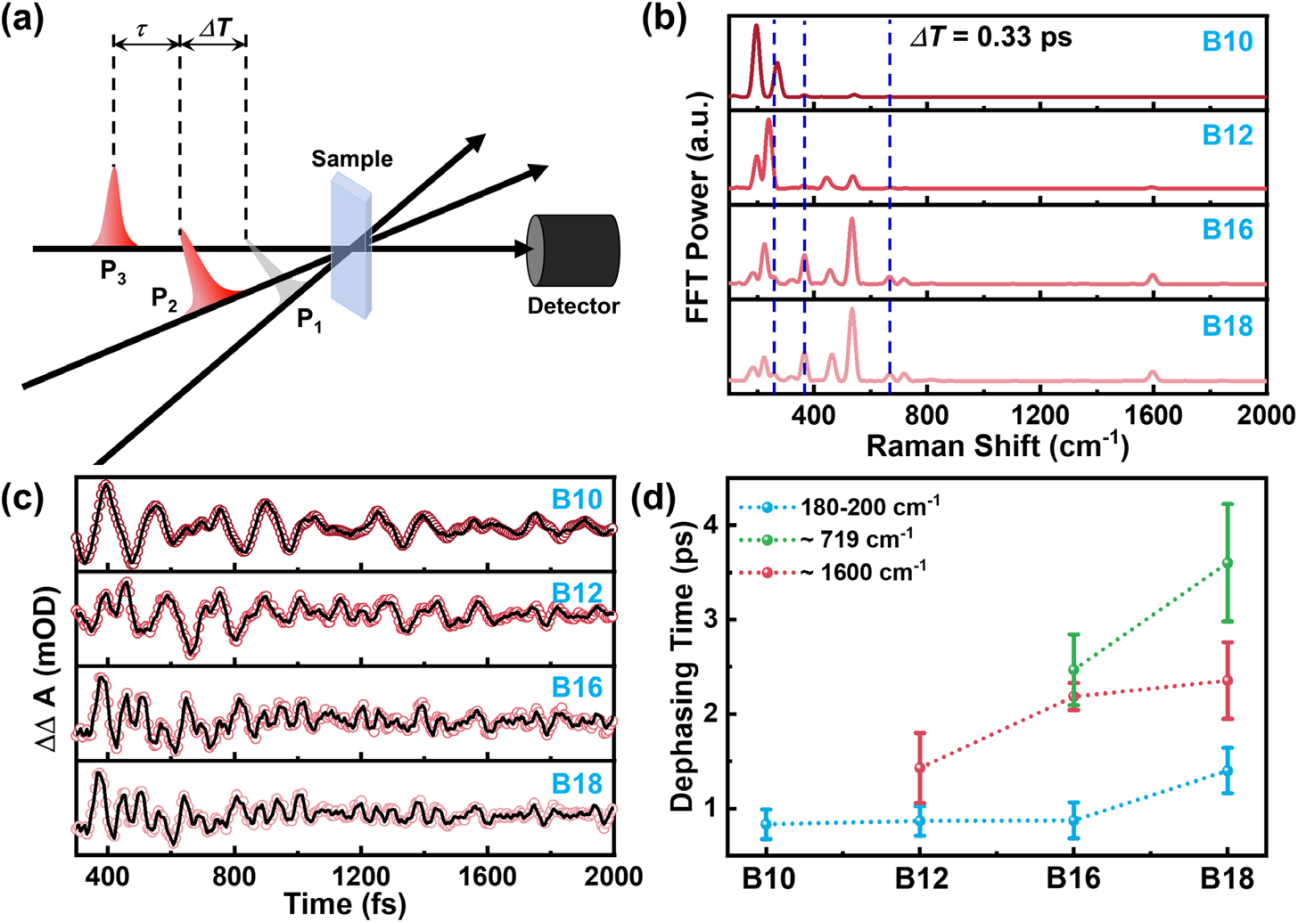
(a) Schematic diagram of TR‐ISRS. P_1_, P_2_, and P_3_ stand for the first, second and third pulses. (b) The excited‐state Raman spectra of the PBI derivatives at *ΔT* of 0.33 ps. The blue dotted lines denote the vibrational signal of CHCl_3_ [23]. (c) The oscillatory signal fitting of the PBI derivatives. (d) The dephasing time comparison across the PBI derivatives with the data collected at *ΔT* of 0.33 ps, 5 ps, and 100 ps, respectively.

To directly probe the excited‐state structural dynamics governing nonradiative relaxation in PBI derivatives, TR‐ISRS [23, 24, 25] measurements were performed (Figure 4a). Following the scheme used in the fs‐TA experiments, an actinic pump pulse (P_1_, 535 nm) was employed to generate the excited‐state population, and a time‐delayed NIR Raman pump pulse (P_2_, sub‐10 fs; Figure S12), resonant with the ESA band in the 680–850 nm region (Figure S9), was used to impulsively excite coherent vibrational motion via resonant Raman scattering. A broadband Raman probe pulse (P_3_, sub‐10 fs; Figure S12) then monitored the induced differential absorption as a function of probe delay time (*τ*). The transient signals contain both population dynamics and vibrational coherence contributions (Figure S13a). After fitting the population kinetics with a multiexponential decay model, subtraction of the population component yields oscillatory residuals corresponding to coherent vibrational motion (Figure S13b), whose Fourier transformation provides the excited‐state Raman spectra (Figure S14).

The resulting spectra exhibit two well‐defined frequency regions (Figure 4b): a low‐frequency region below 1000 cm^−1^ and a high‐frequency band centered near 1600 cm^−1^. A detailed assignment is summarized in Table S3, with the distribution of low‐frequency modes further visualized in Figure S15. The low‐frequency region is dominated by collective skeletal deformations, ring‐breathing modes, and out‐of‐plane distortions (Figure S16) [35, 36], corresponding to soft structural coordinates involving large‐amplitude nuclear motion. In contrast, the high‐frequency band near 1600 cm^−1^ arises from aromatic C═C stretching within the conjugated PBI framework (Figure S17) [23, 33], reflecting vibrational motion strongly coupled to the delocalized π‐electronic structure and therefore highly sensitive to structural distortion.

Pronounced bending‐dependent changes are observed in both the spectral distribution and temporal persistence of the vibrational features. In the most strongly bent derivative **B10**, the excited‐state Raman response is dominated by only a few low‐frequency modes (notably near 196 and 271 cm^−1^), while the high‐frequency aromatic band is essentially absent within the experimental time window. For **B12**, the high‐frequency mode near 1600 cm^−1^ becomes weakly discernible but remains significantly attenuated, and the low‐frequency pattern is already simplified. In contrast, the less distorted derivatives **B16** and **B18** exhibit a prominent and persistent high‐frequency band together with a richer set of resolved low‐frequency modes. Thus, increasing structural bending leads to suppression of the aromatic skeletal vibration and progressive loss of resolved low‐frequency features.

The systematic evolution shown in Figures 4b and S15 is particularly revealing. As the molecular framework bends from **B18** and **B16** to **B12** and **B10**, the number of resolved low‐frequency modes decreases, while the high‐frequency aromatic stretching mode weakens and ultimately disappears. This behavior does not indicate the absence of low‐frequency structural motion in the strongly bent derivatives. Rather, it suggests that the motions undergo accelerated dephasing following photoexcitation, such that their coherent signatures are no longer spectroscopically observable [37, 38, 39]. The attenuation of the aromatic C═C stretching mode further indicates that vibrational coherence associated with the electronically delocalized framework is progressively destabilized with increasing distortion. Together, the observations indicate that bending transforms the excited‐state vibrational manifold into a more dissipative environment in which coherence is rapidly lost and energy redistribution is enhanced.

Quantitative analysis of vibrational dephasing dynamics (Figure 4c) provides further support for this interpretation. The extracted dephasing times are summarized in Table S4. In the more strongly bent derivatives, many vibrational modes become too short‐lived to be spectroscopically resolved, such that the measured dephasing times reflect only the subset of modes that remain observable. Among modes that can be directly compared, the low‐frequency skeletal deformation mode near 180–200 cm^−1^ exhibits a clear bending dependence. Its dephasing time decreases from 1401 fs in **B18** to 873 fs in **B16**, 868 fs in **B12**, and 832 fs in **B10** (Figure 4d), indicating progressively faster loss of coherence with increasing distortion. Given that this mode corresponds to a soft structural coordinate involving large‐amplitude deformation of the PBI framework and tether, its accelerated dephasing reflects enhanced structural fluctuation and more efficient vibrational energy redistribution.

A similar trend is observed for the high‐frequency aromatic C═C stretching mode. This mode remains well‐resolved in **B18** and **B16**, with long coherence lifetimes of 2354 and 2186 fs, respectively, but shortens to 1429 fs in **B12** and becomes undetectable in **B10**. Additional support is provided by the mode near 719 cm^−1^, which is clearly resolved with long dephasing times in **B18** and **B16** but disappears in **B12** and **B10**. Collectively, the results demonstrate that structural bending destabilizes both delocalized aromatic vibrational coherence and a substantial fraction of low‐frequency structural coherences. Accordingly, the excited‐state vibrational manifold evolves from a coherent, mode‐rich response in weakly distorted PBI derivatives to a rapidly dephasing, dissipative landscape in strongly bent derivatives.

## 3 Discussion

**SCHEME 1:**
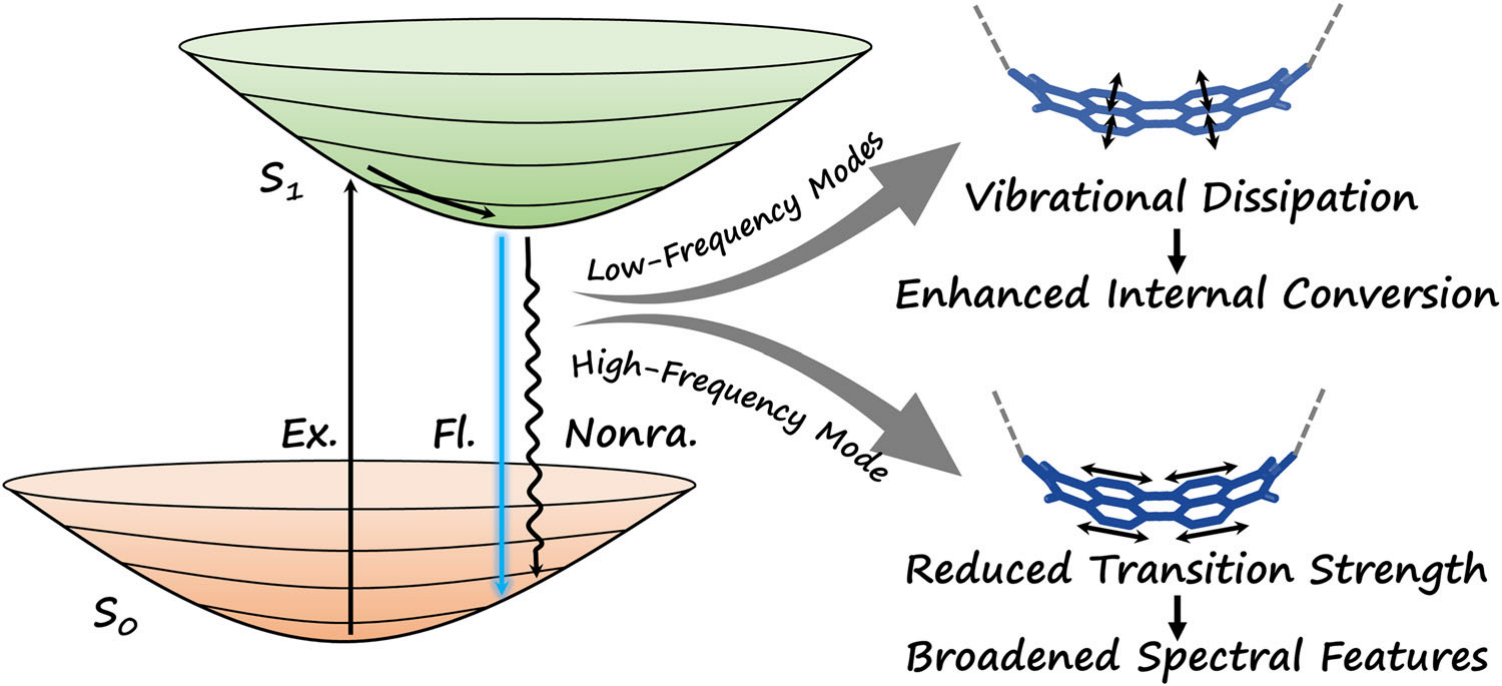
Schematic diagram of bending‐induced reorganization of vibrational landscape. Ex., Fl., and Nonra. are short for excitation, fluorescence and nonradiative decay, respectively.

The mechanistic roles of the two Raman frequency regions can be more clearly delineated (Scheme 1). The high‐frequency band near 1600 cm^−1^, arising from aromatic C═C stretching within the PBI framework, provides a sensitive probe of the coherent vibrational response of the aromatic core in the excited state. Within the *Albrecht* formalism of resonance Raman scattering [40, 41], the intensity of a vibrational mode is governed not only by the mode‐specific nuclear displacement but also by electronic transition contribution, which is represented in the present semi‐quantitative analysis by the calculated transition dipole moment. Accordingly, variations in Raman intensity cannot be interpreted solely in terms of the magnitude of overall structural distortion.

In this context, the attenuation of the 1600 cm^−1^ band with increasing molecular bending cannot be attributed exclusively to enhanced structural reorganization. Although the root‐mean‐square deviation (RMSD) between S_0_ and S_1_ geometries (Figure S18) provides a qualitative measure of structural distortion, its variation does not correlate with the experimentally observed decrease in Raman intensity. Instead, the mode‐specific Raman analysis (detailed in the Supporting Information) further shows that **B10** exhibits the lowest simulated relative Raman response for both selected aromatic C═C stretching modes associated with the high‐frequency band (Table S5). Notably, the higher‐frequency modes display a progressive increase in simulated response from **B10** to **B18**. It means that the attenuation of the high‐frequency Raman response upon strong bending is consistent with the combined contributions of mode‐specific displacement along the aromatic C═C stretching coordinates and diminished electronic‐transition‐mediated Raman enhancement, rather than simply following the magnitude of overall structural reorganization.

Consistent with the mode‐specific analysis described above, the attenuation of the ∼1600 cm^−1^ aromatic C═C stretching mode from the nearly planar to the increasingly bent PBI derivatives reflects a systematic reduction in resonance Raman intensity arising from both decreased mode‐specific displacement and diminished electronic‐transition‐mediated Raman enhancement. Previous studies based on *Albrecht* theory and resonance Raman spectroscopy have established that Raman intensities are governed by the interplay between vibrational displacement along the relevant normal coordinate and the strength of the associated electronic transition [40, 42, 43, 44, 45]. In the present series, increasing structural bending is accompanied by a progressive decrease in the calculated transition dipole moments, which directly contribute to the resonance of Raman cross‐section. Concurrently, time‐domain Raman measurements reveal a systematic shortening of the vibrational coherence lifetime of the ∼1600 cm^−1^ mode. The reduction in Raman intensity and the acceleration of vibrational dephasing therefore occur in parallel as the degree of bending increases. These observations suggest that structural bending not only weakens the resonance‐enhanced vibrational response associated with the aromatic C═C stretching coordinate but also promotes more rapid loss of vibrational phase coherence. Together, these effects indicate that bending‐induced reorganization of the excited‐state vibrational landscape modifies both the amplitude and persistence of coherent vibrational motion, providing a mechanistic link between structural distortion and enhanced nonradiative energy dissipation.

In contrast, the low‐frequency modes probe collective vibrational coordinates of the geometrically constrained molecular framework, and their coherence dynamics provide a sensitive window into the evolution of the excited‐state vibrational landscape [39, 46], which offers direct insight into how structural distortion influences vibrational relaxation processes. The progressive loss of resolved low‐frequency Raman peaks, together with the shortened dephasing times of representative modes, indicates that the low‐frequency vibrations become increasingly difficult to sustain as molecular bending increases. While the reduced number of observable modes may partially reflect weakened Raman response and decreased Raman cross sections, this effect alone cannot account for the observed dynamical behavior. More importantly, quantitative analysis of the time‐domain response reveals that the extracted dephasing times decrease systematically with increasing structural distortion. This monotonic trend provides direct and unambiguous evidence that vibrational coherence decays more rapidly in the bent derivatives. The result is mechanistically significant, as low‐frequency modes provide efficient channels for dissipating electronic energy through collective nuclear motion within the singlet manifold [6, 30]. Accelerated dephasing of these modes therefore signifies enhanced vibrational energy redistribution and increased efficiency of nonradiative decay.

To further clarify the physical origin of accelerated dephasing, we consider how structural bending reshapes the excited‐state vibrational landscape. In the vibrational dephasing model proposed by Shelby et al. [47], dephasing in polyatomic molecules can arise from frequency fluctuations generated by anharmonic coupling between the observed vibration and low‐frequency bath modes. In the present PBI series, increasing structural bending does not necessarily imply greater conformational flexibility. Rather, shortening the tether imposes stronger geometric constraints while simultaneously introducing greater structural distortion into the chromophore. Within this framework, structural bending in the present system can reorganize anharmonic coupling pathways involving collective framework deformation modes, aromatic skeletal modes, and tether‐related motions in the excited‐state vibrational response. Such bending‐induced reorganization can enhance the dynamic modulation of coherent vibrational frequencies by low‐frequency structural motions, thereby accelerating the loss of vibrational phase coherence. Thus, the accelerated dephasing observed here should not be viewed simply as a consequence of increased conformational flexibility, but rather as being consistent with bending‐induced reorganization of anharmonic coupling pathways involving low‐frequency structural motions and the resulting dynamic modulation of coherent vibrational frequencies.

Taken together, the excited‐state Raman results establish a direct spectroscopic connection between molecular bending and reorganization of the excited‐state vibrational landscape. Increasing distortion attenuates the high‐frequency aromatic stretching response while simultaneously reducing both the number and lifetime of coherent low‐frequency vibrations. Dephasing analysis further reveals that even the remaining modes lose coherence more rapidly with increasing bending. Importantly, while variations in electronic‐transition‐mediated Raman enhancement can influence the visibility and apparent intensity of vibrational modes, the systematically shortened coherence lifetimes obtained from quantitative time‐domain fitting provide the most direct evidence that accelerated vibrational dephasing is intrinsically induced by structural bending. Rather than reflecting a simple reduction of the vibrational complexity, this observed spectral evolution signifies the emergence of a more dissipative excited‐state environment in which vibrational coherence is rapidly lost and energy redistribution is facilitated. In this framework, the concurrent weakening of high‐frequency Raman response and acceleration of vibrational dephasing together define the restructuring of the excited‐state vibrational landscape under bending. Accelerated vibrational dephasing observed with increasing structural bending provides direct dynamical evidence that nonradiative energy dissipation is governed by bending‐induced reorganization of the excited‐state vibrational landscape. By modifying anharmonic coupling pathways and facilitating more efficient intramolecular vibrational energy redistribution, structural distortion promotes faster vibrational coherence loss and thereby enhances nonradiative decay. These findings establish a mechanistic connection between molecular deformation, vibrational dynamics, and energy dissipation in electronically excited chromophores. This dynamic picture is fully consistent with the steady‐state and time‐resolved photophysical observations and provides direct spectroscopic evidence that bending‐enhanced nonradiative decay originates from vibrational landscape reorganization that promotes efficient IC within the singlet manifold.

Although the present study employs bent PBIs as a structurally well‐defined model system, the underlying mechanism revealed here is expected to be relevant to a much broader class of structurally distorted π‐electronic chromophores. Molecular deformations such as bending, twisting, curvature, folding, or conformational constraint can perturb electronic transition properties, redistribute vibronic interactions, and reorganize excited‐state vibrational coupling pathways. Consequently, the resulting modification of vibrational coherence and energy redistribution processes may substantially influence nonradiative relaxation dynamics. In this context, curved nanographenes, helicenes, cycloparaphenylenes, bowl‐shaped aromatic systems, and twisted donor–acceptor chromophores may represent particularly promising platforms for exploring analogous structure–vibrational dynamics relationships. More broadly, the present results suggest that structural deformation should not merely be viewed as a geometric perturbation of molecular structure, but rather as a means of engineering the excited‐state vibrational landscape itself. Such “vibrational landscape engineering” may provide a general strategy for controlling energy dissipation pathways and excited‐state dynamics in functional molecular materials. Complementary time‐resolved vibrational techniques could further elucidate the microscopic origins of these effects. For example, time‐resolved infrared (TRIR) spectroscopy [48, 49] is capable of monitoring local bond‐specific structural evolution, whereas TR‐ISRS directly resolves coherent vibrational wave packet motion and dephasing dynamics. Complementary ultrafast Raman techniques, such as femtosecond stimulated Raman spectroscopy [50, 51] (FSRS) and multidimensional Raman spectroscopy [52, 53], may provide additional insight into vibrational coupling and energy redistribution processes. Future studies integrating these approaches may therefore provide a more comprehensive picture of how structural deformation governs vibrational energy flow, coherence loss, and nonradiative energy dissipation in electronically excited molecular systems.

## 4 Conclusion

In summary, this work establishes a direct and mechanistic link between molecular geometry and excited‐state energy dissipation by systematically interrogating a series of PBI derivatives with controlled structural bending. The combined steady‐state and time‐resolved spectroscopic analyses reveal a clear and nontrivial trend: increasing distortion suppresses radiative emission while markedly accelerating nonradiative decay within the singlet manifold, with negligible contribution from ISC. Crucially, time‐resolved Raman measurements provide direct experimental access to the underlying structural dynamics, demonstrating that bending attenuates the high‐frequency aromatic skeletal vibrational response, redistributes the low‐frequency vibrational manifold, and induces rapid vibrational dephasing.

The findings herein uncover a previously underappreciated mechanism of nonradiative relaxation in π‐conjugated systems. Rather than being governed primarily by energy‐gap modulation, bending‐driven nonradiative decay originates from the reorganization of the excited‐state vibrational landscape that creates a highly dissipative environment for electronic energy relaxation. This work therefore identifies vibrational dissipation, mediated by structural distortion, bending‐induced reorganization of vibrational coupling pathways, and the resulting coherence loss, as a central factor controlling IC.

Beyond resolving a long‐standing mechanistic ambiguity, the present study introduces a general structure–vibrational dynamics paradigm for understanding and controlling excited‐state relaxation in conjugated chromophores. The ability to modulate vibrational coherence and energy redistribution through molecular geometry provides a new and powerful design principle for tuning energy dissipation pathways. The insights have broad implications for the rational design of functional organic materials, including optoelectronic devices, light‐harvesting systems, and molecular photonic platforms, where precise control over excited‐state energy flow is essential.

## Conflicts of Interest

The authors declare no conflicts of interest.

## Supporting information



The authors have cited additional references within the Supporting Information [21, 42, 54, 55, 56, 57, 58, 59, 60]. **Supporting File 1**: anie73330‐sup‐0001‐SuppMat.docx.

## Data Availability

The data that supports the findings of this study are available in the Supporting Information of this article.
