# Supplementary material for: Bending‐Induced Vibrational Landscape Reorganization Governs Energy Dissipation in Perylene Bisimides
Source: Angew Chem Int Ed Engl. 2026 Jun 26;65(35):e1926167. doi: 10.1002/anie.1926167 (PMC13502608; doi:10.1002/anie.1926167)
Supplement: Supplementary file 1 [file ANIE-65-e1926167-s001.docx]

***Supporting Information***

**Bending-Induced Vibrational Landscape Reorganization Governs Energy Dissipation in Perylene Bisimides**

Wei Zhang,^a#^ Di Zhao,^b#^ Byeongjoo Kang,^c#^ Hui-Jun Zhang,^b^ Yeonju Park,^d^ Young Mee Jung,^d^ Woojae Kim,^c*^ Jianbin Lin,^b*^ Dongho Kim^a*^

^a^Spectroscopy Laboratory for Functional π-Electronic Systems and Department of Chemistry, Yonsei University, Seoul 03722, Republic of Korea.

^b^Department of Chemistry, College of Chemistry and Chemical Engineering, MOE Key Laboratory of Spectrochemical Analysis and Instrumentation, Xiamen University, Xiamen 361005, P. R. China.

^c^Department of Chemistry, Yonsei University, Seoul 03722, Republic of Korea.

^d^Department of Chemistry, Institute for Molecular Science and Fusion Technology, and Kangwon Radiation Convergence Research Support Center, Kangwon National University, Chuncheon, 24341, Republic of Korea.

^#^These authors contributed equally.

E-mail: [woojae@yonsei.ac.kr](mailto:woojae@yonsei.ac.kr) (W. K.), [jb.lin@xmu.edu.cn](mailto:jb.lin@xmu.edu.cn) (J. L.), [dongho@yonsei.ac.kr](mailto:dongho@yonsei.ac.kr) (D.K.)

**S1. Materials and Methods**

**1. Materials**

The synthesis of PBI derivatives was reported previously.^[1]^ The chemicals CHCl_3_ and **Ref** were bought from Sigma-Aldrich, direct used with no further purification.

**2. Theoretical Calculations**

Structural optimization and Raman simulation were performed based on the DFT and TD-DFT by employing the Gaussian 16, Revision C.02 package.^[2]^ Optimization of the S_0_ state geometries were obtained using the wb97xd^[3]^ functional at the basis set def2SVP^[4]^ level. The FMOs and electron-hole analysis were performed by using the Multiwfn^[5]^ and VMD^[6]^ software based on the outputs from Gaussian. The Raman vibrational modes were drawn by using the VMD. The RMSD was calculated by using VMD.

**3. Quantification of the Structural Bending by Radius of Curvature**

To provide a quantitative metric for the degree of structural bending, the curvature radius of the PBI core was estimated from the optimized ground-state geometries. The radius of curvature was selected because it provides a simple geometric descriptor of global bending: a smaller radius corresponds to a more strongly curved structure, whereas a planar or nearly planar structure gives a very large curvature radius.

Three representative atoms (Figure S2) along the long axis of the PBI core were selected to define the curvature circle. Specifically, the two terminal imide nitrogen atoms were used as the two end points of the PBI framework, and one carbon atom located in the central benzene unit along the long-axis direction was selected as the central point. The three atoms were chosen because they lie along the principal bending direction of the PBI core and provide a consistent descriptor of the overall curvature across the molecular series.

The radius of curvature was calculated as the circumradius of the triangle defined by the three selected atoms. The three side lengths were denoted as *a*, *b*, and *c*. The semiperimeter *L* and triangle area *S* were calculated using Heron’s formula:

$L=\frac{a+b+c}{2}$	(S1)

$S=\sqrt{L\left( L-a \right)\left( L-b \right)\left( L-c \right)}$	(S2)

The curvature radius *R* can be obtained following:

$R=\frac{abc}{4S}$	(S3)

**4. Steady-State Absorption and Emission**

The optical absorption spectra were recorded with JASCO V-770 spectrometer at room temperature.

Fluorescence spectra were carried out on a F-7000 spectrometer (Hitachi). Fluorescence spectra are spectrally corrected by using correction factor of the fluorescence spectrophotometer.

The PLQYs were measured by applying the comparative method with rhodamine-6G as the reference (solvent: ethanol, excited at 488 nm, the PLQY of rhodamine-6G is 94%).^[7]^

The solution concentrations used in this work are 10^-5^ mol/L.

The error bars of *k_r_* and *k_nr_* were calculated based on following equations:

$\Delta k_{r}=k_{r}\sqrt{{(\frac{\Delta\Phi_{PL}}{\Phi_{PL}})}^{2}+{(\frac{\Delta\tau}{\tau})}^{2}}$	(S4)

$\Delta k_{nr}=k_{nr}\sqrt{{(\frac{\Delta\Phi_{PL}}{1-\Phi_{PL}})}^{2}+{(\frac{\Delta\tau}{\tau})}^{2}}$	(S5)

**5. Steady-State Raman Measurements**

Prior to Raman measurements, a 2 μL droplet of the sample solution (dissolved in dichloromethane) was deposited onto a Si wafer and allowed to dry before each measurement. Raman spectra were acquired using a Raman spectrometer (XPER-C, Nanobase, Korea) with a 473/785 nm excitation source. The exposure time was set to 0.5 s, and each spectrum was accumulated 20 times. All Raman measurements were repeated at least five times to ensure reproducibility.

**6. Ns-Time Resolved PL Spectra**

The ns-time resolved PL spectra were measured with an ICCD detector (PI-MAX4, Princeton Instruments) after photoexcitation using 400 pump (170 fs) generated through an optical parametric amplifier (OPA, ORPHEUS, Light Conversion) and its second harmonic (SH, LYRA-SH, Light Conversion) module, pumped by a Yb:KGW amplifier (PHAROS-SP-1.5mJ, Light Conversion, 1030 nm, 600 μJ, 10 kHz, 176 fs).

**7. Fs-TA**

The setup is driven by a Ti:sapphire regenerative amplifier system (800 nm, 350 μJ, 35 fs, Spitfire Pro, Spectra Physics) operating at a 10 kHz repetition rate. This amplifier pumps an optical parametric amplifier (TOPAS C, Spectra Physics), which generates tunable pump pulses with a pulse width of approximately 100 fs across a broad spectral range of 280-2700 nm. The pump wavelength is 535 nm. A small portion of the fundamental 800 nm beam is split off before the OPA and focused onto a 4 mm thick sapphire window to produce a broadband white light continuum, which serves as the probe beam. The time delay between the pump and probe pulses is precisely controlled by a variable optical delay stage (ILS300LM-S, Newport). For signal detection, the pump beam is chopped at 500 Hz, and the transient absorption difference is calculated by alternately measuring the absorption spectra with and without the pump pulse. At each delay time, 4000 pulses are co-added to ensure a high signal-to-noise ratio. The probe intensity is monitored by a High S5 Speed Spectrometer (Ultrafast Systems). To eliminate polarization-dependent signals, the angle between the pump and probe beams is set to the magic angle (54.7°).

**8. Ns-TA**

Ns-TA experiments were performed using a commercial spectrometer (EOS, Ultrafast Systems). The excitation pulses were generated by a commercial collinear optical parametric amplifier (ORPHEUS, Light Conversion) combined with a second-harmonic generation stage (LYRA-SH, Light Conversion). The Yb:KGW regenerative amplifier (PHAROS-SP-1.5mJ, Light Conversion, 1030 nm, 600 μJ, 10 kHz, 176 fs) was used as the main source for ns-TA.

**9. TR-ISRS**

A Yb:KGW regenerative amplifier (PHAROS-SP-1.5 mJ, Light Conversion, 1030 nm, 600 μJ, 10 kHz, 176 fs) was used as the main source for TR-ISRS. The fundamental pulse (1030 nm) passes through a periscope and then it is divided into two pulses by 7(R):3(T) beam splitter. A smaller portion of the fundamental pulse generates actinic pump (P_1_, 535 nm, ~ 170 fs) by a commercial collinear optical parametric amplifier (ORPHEUS, Light Conversion) combined with a second-harmonic generation stage (LYRA-SH, Light Conversion). A larger portion of the fundamental pulse passes through a periscope and is additionally divided into two pulses by a 1(R):9(T) beam sampler to generate a home-built noncollinear optical parametric amplifier (NOPA). A white-light seed pulse (from reflected pathway, generated in a 2-mm-thick BBO crystal (type 1, theta = 23.4°, Eksma optics)) and a second harmonics (from transmitted pathway, 515 nm) are spatially and temporally overlapped in a 2-mm thick BBO crystal (type 1, theta = 24°, Eksma optics), which generates a broadband pulse covering the near-infrared region (700-900 nm, Fig. S8a). Pulse compression of the NIR-NOPA output is carried out by using a pair of chirped mirrors (#108243, Layertec) and wedges (23RQ12-02-M). We carefully determined the number of bounces (7 bounces/mirror) on the chirped mirrors and thickness at certain position of the wedges considering additional chirps after the pulse compression stage (by a half-wave plate, a neutral density filter, a beam splitter and a sample window). The pulse compressed NIR-NOPA output is estimated to be <10 fs, which is retrieved by second harmonic generation frequency resolved optical gating (SHG-FROG) in a 10 μm BBO crystal (type 1, theta = 28°, Eksma optics) (Figure S8b). To prepare Raman pump (P_2_) and probe (P_3_) pulses, the compressed NIR-NOPA is divided into two by a 1(R):1(T) beam splitter (Venton). In particular, we do not need to use additional compensation plate for the reflected pulse owing to specially designed broadband dielectric coating that makes the group delay dispersion (GDD) of the transmitted and reflected pulses identical. The delay times, T (the time interval between the P_1_ and P_2_ pulses) and τ (the time interval between the P_2_ and P_3_ pulses), are controlled by the motorized linear stages (M-ILS250HA (Newport) and M-VP-25XA (Newport) for T and τ, respectively). In the probe beamline, a small portion of NOPA reflected from the neutral density filter (variable, reflective-type, NDC-50S-1, Thorlabs) is used as the reference pulse for balanced detection. The P_1_ pulse and the P_2_/P_3_ pulses are focused by a plano-convex lens (f = 250mm) and a concave mirror (f = 200 mm), respectively. At the focal point, beam diameters (1/e^2^) of the P_1_ (535 nm, 300 nJ), P_2_ (800 nm,120 nJ), and P_3_ (800 nm, 2 nJ) pulses are 300-350, 100, and 100 μm, respectively. Polarizations of all three pulses are parallel (horizontally polarized) at the sample position. To minimize GDD, we use an ultrathin wall aperture (200 μm-thick UVFS) flow cell (48/UTWA2/Q/0.2, Starna) with 200 μm optical path length. Also, a micro annular gear pump (mzr-4622 M2.1) with Teflon tubing is used for flowing the sample solutions which can remove the thermal-lens effect due to the 10 kHz repetition rate of the laser and avoiding photo-degradation of the sample. We usually prepare 2–3 mL sample solutions for the experiments. The optical densities at 535 nm for **B10**-**B18** are ~0.6 in the 100 μm path length cell (c = 100 μM). The P_3_ and the reference pulses are detected using the Si photodiodes (S2281-04, Hamamatsu) without the color or band-pass filters for open-band (non-dispersive) detection in order to minimize the vibrational coherences from the ground-state of the sample and solvent. The input signals are first amplified by a low-noise current preamplifier (SR570, Stanford Research Systems) and then the peak voltages of every laser shot are recorded by a data acquisition (DAQ) card (NI-PCI-6220, National Instruments) with setting a sampling rate of 10000/sec. The P_2_ pulse is modulated at 5 kHz by a mechanical chopper (MC1F60, Thorlabs), which allows data processing in a shot-to-shot fashion. Especially, for TA experiments with P_1_ and P_3_ pulses, The P_2_ pulse is blocked by a beam block and the P_1_ pulse is modulated at 5 kHz by a mechanical chopper (MC1F60, Thorlabs) to get P_1_-induced TA signals.

**10. Mode-Specific Raman Response Analysis**

To perform the mode-specific Raman analysis, we conducted a semi-quantitative normal-mode projection analysis using the two dominant aromatic C=C stretching normal coordinates identified in the calculated ground-state Raman spectra, located at approximately 1665 and 1690 cm^-1^ (Figure 3b and Table S2). The calculated normal modes were selected as representative high-frequency aromatic C=C stretching coordinates for evaluating the displacement contribution to the observed Raman-response trend, rather than as exact frequency assignments of the excited-state TR-ISRS band observed experimentally near 1600 cm^-1^.

The theoretical basis of the present analysis follows the *Albrecht* treatment of Raman intensities as implemented for conjugated molecular systems by Heimel et al.^[8]^ In this framework, the total scattered-light intensity for a transition from an initial state $\left| \Psi_{i}\rangle\right.$ to the final state $\left| \Psi_{f}\rangle\right.$ is given by

$I_{if}\propto{(\omega_{0}-\omega_{if})}^{4}\sum_{\rho,\sigma-1}^{3} \left| \left\langle\Psi_{i} | \hat{\alpha}_{\rho,\sigma} | \Psi_{f} \right\rangle\right|^{2}$	(S6)

Equation S6 is reproduced using the notation shown in ref. [8]. Here, the $\omega_{0}$ is the energy of the incident photons, $\omega_{if}$ is the transition energy between the initial and final states, and $\hat{\alpha}_{\rho,\sigma}$ denotes a Cartesian component of the polarizability tensor, with $\rho$ and $\sigma$ representing Cartesian directions.

For the first-order Stokes scattering into vibrational mode *a*, the initial state is the electronic and vibrational ground state (*g0*) and the final state is the electronic ground state containing one vibrational quantum in mode *a* (*g1_a_*). Accordingly, $\omega_{if}=\omega_{a}$, where $\omega_{a}$ is the vibrational energy quantum of mode *a*. The corresponding polarizability matrix element is expressed as:

${(\alpha_{\rho\sigma})}_{g0,{gl}_{a}}=A_{a,\rho\sigma}+B_{a,\rho\sigma}$	(S7)

where $A_{a,\rho\sigma}$ and $B_{a,\rho\sigma}$ denote the *Albrecht A*-term and *B*-term contributions, respectively, to the (*ρ*,*σ*) Cartesian component of the polarizability for first-order Stokes scattering into vibrational mode *a*. In the present semi-quantitative analysis, only the *A*-term contribution is adopted to construct a reduced comparative descriptor for the selected high-frequency Raman modes. This approximation is used only to evaluate the relative trend associated with the mode-specific displacement and electronic transition strength; it is not intended to demonstrate that all non-*A*-term contributions are negligible in the experimental TR-ISRS response. Under this approximation:

${(\alpha_{\rho\sigma})}_{g0,{gl}_{a}}\approx A_{a,\rho\sigma}$	(S8)

Substitution into Equation S6 gives:

$I_{a}^{(A)}\propto{(\omega_{0}-\omega_{a})}^{4}\sum_{\rho,\sigma} \left| A_{a,\rho\sigma} \right|^{2}$	(S9)

For comparison of the closely spaced high-frequency aromatic C=C stretching modes across the PBI series, the scattered-frequency prefactor and tensor-dependent proportionality factors are treated as comparable. Therefore, the reduced *A*-term-based relative response can be represented as:

$I_{a}\propto\left| A_{a} \right|^{2}$	(S10)

where $\left| A_{a} \right|^{2}\equiv\sum_{\rho,\sigma} \left| A_{a,\rho\sigma} \right|^{2}$.

The *Albrecht A*-term contribution for first-order Stokes scattering into vibrational mode *a* is given as:^[8]^

$A_{a,\rho\sigma}=2{(\omega_{a})}^{3/2}\sum_{e} \Delta Q_{a}^{e}{(\mu_{\rho})}_{eg}^{0}{(\mu_{\sigma})}_{ge}^{0}\frac{\omega_{eg}^{2}+\omega_{0}^{2}}{{{(\omega}_{eg}^{2}-\omega_{0}^{2})}^{2}}$	(S11)

where the $\omega_{a}$ stands for the vibrational energy quantum of mode *a*, $\Delta Q_{a}^{e}$ is the the contribution of normal mode *a* to the mass-weighted equilibrium geometry change upon electronic excitation from the ground state *g* to excited state *e*, ${(\mu_{\rho})}_{eg}^{0}$ and ${(\mu_{\sigma})}_{ge}^{0}$ are the corresponding electric transition dipole matrix elements, $\omega_{eg}$ is the electronic transition energy, and $\omega_{0}$ is the incident photon energy. Since the vibrational frequencies obtained from the Gaussian calculations are reported as wavenumbers $\tilde{\nu}_{a}$ (in cm^-1^), the $\omega_{a}$ in Equation S11 was converted according to:$\omega_{a}=hc\tilde{\nu}_{a}$	(S12)

where *h* is the Planck’s constant, *c* is the light speed.

For the present PBI series, the high-frequency aromatic C=C stretching response was evaluated under the dominant-transition approximation, in which the lowest relevant electronic excitation is taken as the principal contribution to the Raman response. Under this approximation, and for relative comparison within the molecular series, the *A*-term contribution of mode *a* can be written in reduced form as:

$A_{a}\propto\left( \tilde{\nu}_{a} \right)^{\frac{3}{2}}\Delta Q_{a}\left| \mu_{eg} \right|^{2}F(\omega_{eg},\omega_{0})$	(S13)

where the $\nu_{a}$ is the vibrational frequency expressed in cm^-1^, $\mu_{eg}$ is the calculated transition dipole moment, and

$F\left( \omega_{eg},\omega_{0} \right)=\frac{\omega_{eg}^{2}+\omega_{0}^{2}}{{{(\omega}_{eg}^{2}-\omega_{0}^{2})}^{2}}$	(S14)

represents the resonance-energy contribution.

Combined with Equations S10-S14, the mode-resolved intensity contribution is related to:

$I_{a}\propto\left( \tilde{\nu}_{a} \right)^{3}{(\Delta Q_{a})}^{2}\left| \mu_{eg} \right|^{4}{[F\left( \omega_{eg},\omega_{0} \right)]}^{2}$	(S15)

The dimensional normal-coordinate displacement,$\Delta Q_{a}$, was converted into the corresponding dimensionless displacement, $\Delta_{a}$, according to:^[9]^

$\Delta_{a}=0.172\left( \tilde{\nu}_{a} \right)^{1/2}\Delta Q_{a}$	(S16)

where the $\Delta Q_{a}$ is expressed in amu^1/2^ Å and $\tilde{\nu}_{a}$ is expressed in cm^-1^. Accordingly,

$\left( \tilde{\nu}_{a} \right)^{3}{(\Delta Q_{a})}^{2}\propto\left( \tilde{\nu}_{a} \right)^{2}{\Delta_{a}}^{2}$	(S17)

In our work, the same experimental excitation condition is considered across the PBI series, and the resonance-energy contribution is not used to predict absolute Raman intensities. Instead, a semi-quantitative *Albrecht A*-term-based relative Raman-response descriptor was constructed as:

$I_{a}\propto\left| \mu_{eg} \right|^{4}\left( \tilde{\nu}_{a} \right)^{2}{\Delta_{a}}^{2}{[F\left( \omega_{eg},\omega_{0} \right)]}^{2}$	(S18)

Equation S18 identifies the dependence expected within the reduced *A*-term based treatment.

In the present TR-ISRS experiments, all PBI derivatives were measured under the same Raman pump condition, and their excited-state absorption spectra exhibit closely comparable broad resonance features within the spectral region addressed by the Raman pump. Thus $F\left( \omega_{eg},\omega_{0} \right)$ was treated as an approximately comparable resonance-condition factor and was not explicitly included in the present reduced semi-quantitative descriptor. Besides, the $\tilde{\nu}_{a}$ for each PBI derivative is almost the same, then the $\left( \tilde{\nu}_{a} \right)^{2}$ does not affect the qualitative comparison of the selected mode. To focus on the combined contributions of the calculated transition dipole moment and the mode-specific displacement, a reduced simulated relative Raman-response descriptor was defined as:

$I_{a}\propto\left| \mu_{eg} \right|^{4}{\Delta_{a}}^{2}$	(S19)

The $\mu_{eg}$ and $\Delta_{a}$ can be obtained based on the output from Gaussian. For each PBI derivative, the optimized S_1_/S_0_ geometry difference was projected onto the selected ground-state aromatic C=C stretching normal coordinates to obtain the corresponding dimensionless displacements $\Delta_{a}$. The analysis in here evaluates how strongly the excitation-induced structural reorganization is expressed along the reference high-frequency aromatic stretching coordinates.

We highlight that the Equation S19 is used only as a semi-quantitative descriptor for comparing the relative responses of the selected high-frequency aromatic C=C stretching modes across the PBI series, rather than as a rigorous calculation of the absolute experimental TR-ISRS intensity.

**S2. Supplementary Data**

Figure S1. The optimized geometric conformations of PBIs in ground state (S_0_).

Figure S2. Definition of the three representative atoms used to estimate the curvature radius of the PBI core. The two terminal imide nitrogen atoms and one central carbon atom along the long-axis direction were selected to define an approximate curvature circle.

Table S1. The estimated curvature radius for each PBI derivative.

| sample | *a*/Å | *b*/Å | *c*/Å | *R*/Å |
| --- | --- | --- | --- | --- |
| **B10** | 4.148 | 6.762 | 10.086 | 6.98 |
| **B12** | 4.205 | 6.962 | 10.813 | 10.85 |
| **B16** | 4.261 | 7.124 | 11.375 | 65.74 |
| **B18** | 4.266 | 7.137 | 11.403 | infinite |
| **Ref** | 4.266 | 7.132 | 11.398 | infinite |

Figure S3. Absorption spectra of PBIs plotted in terms of the molar extinction coefficient in CHCl_3_.

Figure S4. The HOMOs and LUMOs based on the optimized conformations in S_0_ state. The isovalue for HOMO and LUMO is 0.02.

Figure S5. The electron-hole analysis of S_1_ state. The isovalue was set as 0.002.

Figure S6. The ns-time resolved PL spectra of PBIs in CHCl_3_. Excited at 450 nm.

Figure S7. The PL lifetimes of PBIs in CHCl_3_.

Figure S8. Dependence of PLQY and *k_nr_* on the S_1_ energy levels. The S_1_ energy levels are shown in Table 1 in the main text.

Figure S9. The fs-TA spectra of PBIs in CHCl_3_. Excited at 535 nm.

Figure S10. The experimental ground-state non-resonant Raman spectra of PBIs with excitation at 785 nm.

Figure S11. The zoom-in steady-state Raman spectra: (a) theoretical simulated spectra, (b) resonant Raman spectra with excitation at 473 nm, (c) non-resonant Raman spectra with excitation at 785 nm. The low-frequency region appears largely featureless under non-resonant excitation because the individual skeletal deformation modes have weak Raman cross sections and are strongly overlapped. Under resonant excitation, the same modes are selectively amplified through vibronic coupling associated with the electronic transition, leading to the emergence of well-resolved low-frequency Raman features

Table S2. The calculated Raman activities of high-frequency aromatic C=C stretching modes.^a^

| samples | Raman activities/Å^4^ amu^-1^ | |
| --- | --- | --- |
|  | 1665 cm^-1^ | 1690 cm^-1^ |
| **B10** | 1831.1637 | 7299.2333 |
| **B12** | 2086.6057 | 8950.7080 |
| **B16** | 2638.1278 | 11494.8508 |
| **B18** | 2723.1348 | 12034.6669 |

a. Raman activities were obtained from Raman simulations (Figure 3b) at the optimized S_0_ geometries. The modes near 1665 and 1690 cm^-1^ are assigned to aromatic C=C stretching vibrations of the PBI framework.

Figure S12. (a) Spectrum of the sub-10 fs NIR-NOPA pulse used for P_2_ and P_3_. (b) SHG-FROG trace of the pulse.

Figure S13. (a) The raw TR-ISRS data of PBIs in CHCl_3_ at particular time delays T and (b) the oscillatory residuals extracted from multiexponential fits to the kinetic traces at each T.

Figure S14. The excited-state Raman spectra of the PBIs. The blue dotted lined denote the vibrational signal of CHCl_3_.

Table S3. The excited-state Raman frequencies comparison of PBIs obtained from TR-ISRS and theoretical simulations (cm^-1^).

|  | **B10** | **B12** | **B16** | **B18** |
| --- | --- | --- | --- | --- |
| Exp. | 196.49, 270.76 | 47.46, 197.36, 241.45, 444.77, 535.71, 1593.38 | 44.97, 182.29, 224.84, 319.77, 457.82, 533.7, 716.80, 1596.38 | 42.47, 184.87, 224.84, 319.77, 457.82, 533.70, 719.49, 1596.38 |
| Theo. | 193.77, 273.63 | 45.72, 196.86, 242.87, 464.19, 523.99, 1598.99 | 46.36, 182.65, 222.05, 311.05, 448.64, 544.50, 715.08, 1598.61 | 46.57, 184.43, 228.30, 318.67, 457.79, 536.29, 714.61, 1601.51 |

Figure S15. The vibration modes (lower than 1000 cm^-1^) comparison across the PBI series.

Figure S16. The theoretical simulated vibrational modes (lower than 1000 cm^-1^) of PBIs in S_1_ state.

Figure S17. The theoretical simulated vibrational modes (around 1600 cm^-1^) of PBIs in S_1_ state.

Table S4. The dephasing time of each mode for the PBIs.

| samples | Wavenumber/cm^-1^ | dephasing time/fs | | |
| --- | --- | --- | --- | --- |
|  |  | *ΔT*=0.33 ps | *ΔT*=5 ps | *ΔT*=100 ps |
| **B10** | 196.49 | 856.6 | 976.7 | 663.0 |
| **B12** | 197.36 | 864.0 | 714.1 | 1026.0 |
|  | 1593.38 | 1509.0 | 1023.2 | 1754.1 |
| **B16** | 182.29 | 894.4 | 672.9 | 1052.1 |
|  | 716.80 | 2879.0 | 2383.1 | 2144.3 |
|  | 1596.38 | 2022.1 | 2256.0 | 2280.0 |
| **B18** | 184.87 | 1644.6 | 1394.1 | 1165.2 |
|  | 719.49 | 3638.1 | 4207.2 | 2144.0 |
|  | 1596.38 | 2734.4 | 1926.1 | 2402.2 |

Figure S18. RMSD values of the PBI derivatives, calculated based solely on the PBI core.

Table S5. Mode-specific displacements and relative response descriptors based on the selected ground-state aromatic C=C stretching reference coordinates of the PBI derivatives.

| sample | *μ_eg_*^a^ | *mode*^b^/cm^-1^ | *Δ_a_* | *I_a_* |
| --- | --- | --- | --- | --- |
| **B10** | 2.954 | 1665 | −0.343 | 8.96 |
|  |  | 1690 | 0.515 | 20.20 |
| **B12** | 3.113 | 1665 | 0.394 | 14.58 |
|  |  | 1690 | 0.620 | 36.10 |
| **B16** | 3.312 | 1665 | 0.411 | 20.33^c^ |
|  |  | 1690 | −0.612 | 45.07 |
| **B18** | 3.368 | 1665 | −0.394 | 19.97^c^ |
|  |  | 1690 | 0.645 | 53.53 |

^a^See Table 1 in the main text. ^b^see Figure 3b and Table S2. ^c^The comparable values for **B16** and **B18** indicate that no strictly monotonic variation is assigned for this individual lower-frequency mode. This minor deviation does not affect the conclusion of the present analysis, because **B10** and **B12** consistently exhibits the lowest simulated response for both high-frequency aromatic C=C stretching modes.

**S3. References**

[1]	R. Wang, B. Qian, Y. Xu, D. Zhao, Q. Chen, Y. Wei, C. Zhang, W. Liang, Y.-B. Jiang, H.-J. Zhang, J. Lin, "Self-Assembled Bent Perylenediimides," *Angew. Chem. Int. Ed.* *64* (2025): e202421871, https://doi.org/10.1002/anie.202421871.

[2]	M. Frisch, G. Trucks, H. Schlegel, G. Scuseria, M. Robb, J. Cheeseman, G. Scalmani, V. Barone, G. Petersson, H. Nakatsuji, e. al., "Gaussian 16, Revision C. 02," *Gaussian, Inc., Wallingford CT* (2019).

[3]	J.-D. Chai, M. Head-Gordon, "Long-range corrected hybrid density functionals with damped atom–atom dispersion corrections," *Phys. Chem. Chem. Phys.* *10* (2008): 6615-6620, https://doi.org/10.1039/B810189B.

[4]	F. Weigend, R. Ahlrichs, "Balanced basis sets of split valence, triple zeta valence and quadruple zeta valence quality for H to Rn: Design and assessment of accuracy," *Phys. Chem. Chem. Phys.* *7* (2005): 3297-3305, <https://doi.org/10.1039/B508541A>.

[5]	T. Lu, F. Chen, "Multiwfn: A multifunctional wavefunction analyzer," *J. Comput. Chem.* *33* (2012): 580-592, <https://doi.org/10.1002/jcc.22885>.

[6]	W. Humphrey, A. Dalke, K. Schulten, "VMD: Visual molecular dynamics," *J. Mole. Graph.* *14* (1996): 33-38, https://doi.org/10.1016/0263-7855(96)00018-5.

[7]	A. M. Brouwer, "Standards for photoluminescence quantum yield measurements in solution (IUPAC Technical Report)," *Pure Appl. Chem.* *83* (2011): 2213-2228, http://dx.doi.org/10.1351/PAC-REP-10-09-31.

[8]	G. Heimel, D. Somitsch, P. Knoll, J.-L. Brédas, E. Zojer, "Effective conjugation and Raman intensities in oligo(para-phenylene)s: A microscopic view from first-principles calculations," *J. Chem. Phys.* *122* (2005): 114511, <https://doi.org/10.1063/1.1867355>.

[9]	G. Orlandi, F. Negri, R. Wilbrandt, F. W. Langkilde, A. M. Brouwer, "Potential energy surfaces of short polyenes in the state T_1_: analysis of time resolved resonance Raman spectra," *Coordin. Chem. Rev.* *125* (1993): 293-300, [https://doi.org/10.1016/0010-8545(93)85026-Z](https://doi.org/https://doi.org/10.1016/0010-8545(93)85026-Z).
